# P-1813. Improving Adherence to an Antibiotic Pre-Authorization Protocol at a Community Academic Medical Center

**DOI:** 10.1093/ofid/ofae631.1976

**Published:** 2025-01-29

**Authors:** Spencer Sutton, Xuping Yan, Richa Tandon, Robert Colgrove

**Affiliations:** Mount Auburn Hospital, Cambridge, Massachusetts; Mount Auburn Hospital, Cambridge, Massachusetts; Mount Auburn Hospital, Harvard Medical School, Cambridge, Massachusetts; Mount Auburn Hospital, Harvard Medical School, Cambridge, Massachusetts

## Abstract

**Background:**

Preauthorization is integral to antimicrobial stewardship and is associated with decreased utilization of restricted antimicrobials, lower cost, and reduced development of antimicrobial resistance. Implementation of such a program is resource-intensive and dependent on the skill of the approving provider. The aim of this quality improvement project was to update the preauthorization program at a community hospital and increase protocol adherence from 23.8% to 85% by September 1st, 2023.

**Figure 1:**
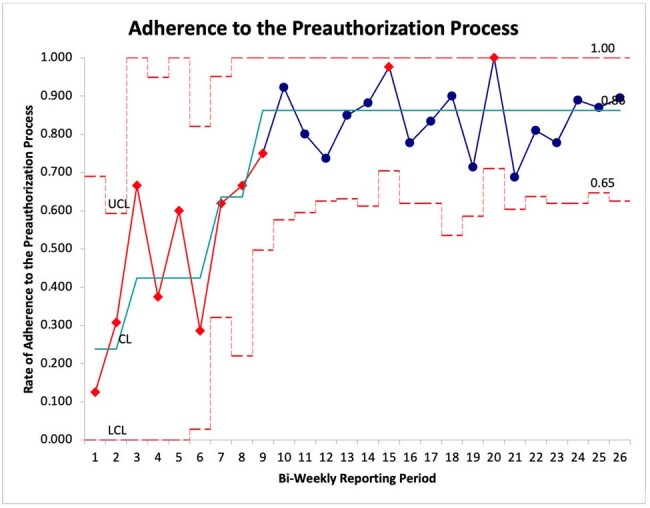
Adherence to the Preauthorization Process presented as a P-chart, representing the rate of the desired outcome with variable sample size over time. Statistical significance identified through mean-line shift, based on 8 consecutive data points above or below a previous mean line.

**Methods:**

A community academic medical center developed a workgroup of infectious diseases (ID) pharmacists and physicians to optimize the existing antibiotic preauthorization policy. Baseline data were collected over a one month period, based on policy requirements for approval prior to implementation of improvement initiatives. Updates implemented an ID pharmacist into the approval process, streamlined request communication, and standardized documentation of approval. Requests were considered adherent to protocol if the process metrics of appropriate use were met. This included following the off-hours pathway appropriately and completion of standardized documentation prior to order verification. Balancing metrics included duration of restricted antimicrobial utilization. Data were extracted through a query of electronic medical records with manual chart review of patients who received at least one dose of a restricted antimicrobial. Data were analyzed through descriptive statistics pursuant to the Institute of Healthcare Improvement’s Model for Improvement or Wilcoxon Rank-Sum, where appropriate.

**Results:**

Between September 2022 and September 2023, 701 restricted therapies were ordered. Of these, 410 were not associated with an infectious diseases consult. Mean protocol adherence increased from 23.8% (5/18) to 86.2% (307/356) [Fig. 1]. Median duration of restricted antimicrobial therapy decreased from 4.0 days to 3.0 days (p=0.0025).

**Conclusion:**

The implemented updates to the preauthorization program markedly increased protocol adherence and decreased duration of restricted antimicrobial therapy. This work demonstrates the feasibility and quality impact of implementing antimicrobial stewardship initiatives in a community hospital setting.

**Disclosures:**

**All Authors**: No reported disclosures

